# New concepts in palliative care in the intensive care unit

**DOI:** 10.5935/0103-507X.20170031

**Published:** 2017

**Authors:** Cristina Bueno Terzi Coelho, James R. Yankaskas

**Affiliations:** 1 University of North Carolina at Chapel Hill - North Carolina, United States.

**Keywords:** Advance care planning, Critical care, Hospice care, Life support care, Palliative care

## Abstract

Some patients admitted to an intensive care unit may face a terminal illness situation, which usually leads to death. Knowledge of palliative care is strongly recommended for the health care providers who are taking care of these patients. In many situations, the patients should be evaluated daily as the introduction of further treatments may not be beneficial to them. The discussions among health team members that are related to prognosis and the goals of care should be carefully evaluated in collaboration with the patients and their families. The adoption of protocols related to end-of-life patients in the intensive care unit is fundamental. A multidisciplinary team is important for determining whether the withdrawal or withholding of advanced care is required. In addition, patients and families should be informed that palliative care involves the best possible care for that specific situation, as well as respect for their wishes and the consideration of social and spiritual backgrounds. Thus, the aim of this review is to present palliative care as a reasonable option to support the intensive care unit team in assisting terminally ill patients. Updates regarding diet, mechanical ventilation, and dialysis in these patients will be presented. Additionally, the hospice-model philosophy as an alternative to the intensive care unit/hospital environment will be discussed.

## Abstract (pt)

Alguns dos pacientes admitidos em uma unidade de terapia intensiva podem enfrentar condições de doença terminal, que geralmente levam à morte. O conhecimento sobre cuidados paliativos é recomendado para os profissionais de saúde encarregados do cuidado destes pacientes. Em muitas situações, os pacientes devem ser avaliados diariamente, já que a introdução de novos tratamentos pode ou não ser benéfica para eles. As discussões entre os membros da equipe de saúde, relacionadas ao prognóstico e aos objetivos do tratamento, devem ser avaliadas cuidadosamente em cooperação com os pacientes e seus familiares. A adoção na unidade de terapia intensiva de protocolos relacionados a pacientes em final da vida é fundamental. É importante ter uma equipe multidisciplinar para determinar se é necessário deixar de iniciar ou mesmo retirar tratamentos avançados. Além disto, pacientes e familiares devem ser informados de que os cuidados paliativos envolvem o melhor tratamento possível para aquela situação específica, assim como respeitar suas vontades e considerar as bases sociais e espirituais dos mesmos. Assim, o objetivo desta revisão foi apresentar os cuidados paliativos como uma opção razoável para dar suporte à equipe da unidade de terapia intensiva na assistência a pacientes com doença terminal. São apresentadas atualizações com relação a dieta, ventilação mecânica e diálise nestes pacientes. Ainda, discutiremos o programa, comum nos Estados Unidos, conhecido como filosofia *hospice*, como alternativa ao ambiente da unidade de terapia intensiva/hospital.

## INTRODUCTION

Any event that precipitates intensive care unit (ICU) admission may lead to irreversible worsening of the symptoms from a chronic disease or an acute event. The multidisciplinary ICU team should continuously re-evaluate the clinical course of their patients, which includes redefining the treatment goals and considering palliative care when there are no benefits to treatment.^(1)^ In some cases, death is inevitable and is being delayed at high psychological, social, and financial costs for all parties involved in this process (patient, family, and health professionals). In many cases, further treatment does not meet the patient's goals of care.^(2)^ These situations are becoming more frequent, as today 20% to 33% of patients die in the ICU.^(3,4)^

What can we do when we are faced with a patient who is on advanced life support but is not presenting any reasonable improvement? What can be offered? In this context, the hospice philosophy fits and has become a program with exponential growth in the United States of America (USA).

In this article, we will discuss the control of symptoms and the role of diet, dialysis, and mechanical ventilation in critically ill patients with terminal illnesses. Finally, it will be stressed how palliative care and a hospice system can be integrated with the ICU team.

## DECISION MAKING

The World Health Organization concludes that only 14% of those in need throughout the world receive palliative care.^(5)^ Many of those patients are treated in ICUs. Due to the great technology available in ICUs for life support, the coexistence of palliative care and intensive care is challenging. Therefore, current critical care should be balanced between palliation and critical curative conditions.^(6-8)^

Additionally, the primary ICU purpose should not only be to promote aggressive treatment; it should also help patients and families make wise end-of-life decisions.^(9-11)^ Therefore, appropriate training is mandatory for intensivists to fulfill this fundamental and current approach.^(2,12)^

Currently, the presence of palliative care service is an accreditation element adopted by the American College of Surgeons Commission on Cancer, and it is used in electing the best American medical centers.^(13^) Palliative care in the ICU supports patients and families and can provide a more comfortable environment, better healing, and increased awareness of the end-of-life.^(14)^

In Brazil, both the legislation and ethical codes have recently changed. The Brazilian Constitution states that human dignity in death are primary rights, and this is aligned with the withdrawal of life support. The law's interpretation assumes that nobody, even in a life-threatening situation, can be forced into medical treatment or surgery.^(15)^ The *Conselho Federal de Medicina* (CFM) Resolution No. 1,805/2006 supports the suspension of futile treatments for the terminally ill's incurable illness if it is accepted by the patient or his legal representative. The advanced directive of will (CFM ordinance no. 1,995/2012) is a legal and ethical document that allows health professionals to respect the will of the person. This directive permits the person to make his own choices on future treatments, such as receiving or refusing treatment if the person is unable to communicate or express his will.

Recently, this topic has raised the attention of the Brazilian medical community. Several national and international publications on palliative care and terminal illness in the ICU have been published.^(16-22)^

## COMMUNICATION

There is evidence that the ICU communication between staff and patients/family members is inadequate. Recent studies have shown that families are dissatisfied with this communication.^(23,24)^ It has been suggested that the quality of this communication is directly related to the family's satisfaction with treatment.^(25,26)^

Patient and family communication can be difficult in the ICU because of illness severity, medical complications, high risk of death, and limited family medical knowledge. Discussions about advanced directives and goals of treatment in hospitals are not carried out frequently by intensivists. Efforts to improve the quality and quantity of these discussions (when the patient is stable) improve the efficiency of the ICUs, reduce the burden of patient care during the end-of-life period, and reduce the burden on the families and health care providers.^(27)^ Some intensivists hesitate to communicate when the problems, treatment options, and prognoses are not well defined. There is limited research on family interactions in the ICU. A systematic approach to daily communication can be effective in these settings. The following approach has been effective in several major U.S. medical centers.

Good communication is an essential part of medical practice in the ICU. Standard practices provide a basis for improving patient care in most settings. The key elements are identifying consistent medical and family (usually the medical decision maker) individuals; setting a regular time for daily meetings; defining the major problems initially and as the clinical course proceeds; identifying and respecting the patient's care preferences; and communicating concisely and consistently.

After the patient's initial assessment and stabilization, the first family meeting is essential to meet the family members, identify the key decision maker, report the initial assessment and diagnostic and treatment plans, assess the existence of advanced directives, and plan subsequent meetings. Many individuals have not had experience with critically ill family members. It is useful to explain the organization and care patterns of the ICU and to explain the roles and hierarchy of the care team. Scheduled meetings, e.g., just after morning rounds, are useful for all. An identified and consistent communicator for each patient can provide reduced ambiguity and confusion. The first meeting is usually the longest and sets the expectations for later meetings. A problem-oriented approach, including resolving, worsening, and new issues, organizes the meetings. Some units encourage family attendance at work rounds. This permits direct observation of the patient interactions and an opportunity to hear the discussions of evaluation and care options. Advance care directives should be identified at the first meeting. If there are no such directives, the family can be advised to consider what their relative might choose if they were fully cognizant of the circumstances. Effective communication can facilitate ongoing care.^(28)^

Clinical progress in the ICU can focus subsequent discussions on status, future care needs, or palliative care options. Families appreciate knowing that their loved one received the best-indicated care options and that their care preferences were respected. This approach is effective whether the outcome is complete recovery, death in the ICU, or transition to palliative care.

The definition of dying with dignity recognizes unconditional intrinsic human values, such as physical comfort, quality of life, autonomy, meaning, preparation, and interpersonal connection. Preserving dignity, avoiding harm, and preventing and resolving conflicts are the responsibilities of the health care provider who is in charge of the patient during the end-of-life.^(8)^ There is a paradigm shift as the focus is on relief of the sick person's suffering instead of on the person's disease.

A common therapeutic approach should be defined based on the previous discussions among the ICU's multidisciplinary team, including the primary care staff and the different specialties that are taking care of the patient. This will avoid uncertain feelings for patients and families. As multiple specialties are frequently involved, such as clinicians, surgeons, oncologists, intensivists, and palliative care, the risk of misunderstanding is great.

This proactive approach to communicating palliative care to patients and families decreased the time of ICU stay and hospitalization and the time lapse between knowledge of the prognosis and the decision to move toward a "Comfort Care" treatment. Comfort care emphasizes the quality of the last days of life over its quantity.^(29,30)^ Family meetings should be based on trust and a clear understanding that the withdrawal or withholding of life support is not related to the withdrawal or withholding of care.^(31)^

Cook et al. observed that the decision to perform mechanical ventilation withdrawal was based on a patient's preference not to use life support, low expectation of survival, and risk of being discharged from the ICU with a high probability of developing low cognitive function.^(32)^ The advanced life support withdrawal should be carried out with the involvement of the multidisciplinary team and family to make sure that this decision is consistent with the values and objectives of that particular patient.^(33)^ After discussing this decision with the patient and family, it must be assured that the entire process will be supervised. Every effort will be made to avoid discomfort, to maintain analgesia, and to control symptoms, such as pain, agitation, and dyspnea.^(8)^

When discussing withholding or withdrawing therapies with the patient and families, the health professional should make it clear that they will continue to provide supportive treatment to them.

Spirituality is a vital part of human wholeness and plays an important role in the healing process. Recent data suggests that spiritual concerns are common in patients with severe disease and most patients want to discuss spirituality with their doctors.^(34-36)^ However, less than 50% of doctors believe that they should address this issue, and only the minority of patients reported that their spiritual needs were assessed.^(37)^ An important topic related to communication is the understanding of the patients' and families' spiritual beliefs. Actively dying patients need special attention to their psychosocial and spiritual needs. Although we cannot offer the hope of a cure, we can offer the hope of a dignified death. There is always something more you can do to comfort the patient and family, no matter how difficult the situation.

Chaplains in the USA have expertise that is complementary to that of health care professionals and provide spiritual support to people of all faith traditions; they also explore spiritual questions and concerns that may arise during hospitalization. Chaplains help people address issues of fear, loneliness, ethical values, questions of meaning, and hope.

## SYMPTOM MANAGEMENT

Opioids are still the main option for pain management in critically ill patients.^(38)^ Intensivists should be prepared to medicate patients before performing procedures such as chest drainage and the withdrawal of introducers; even routine procedures, such as bathing and changing positions, can be very painful for many patients.^(39)^

Dyspnea is treated by optimizing the treatment of the underlying disease, such as using diuretics and inotropic agents for heart failure, providing intravenous hydration suspension, and offering non-drug therapy use. Opioids are the drugs of choice for dyspnea at the end-of-life, as well as dyspnea refractory to the treatment of other diseases. The use of anxiolytics can be useful for reducing dyspnea's anxiety component. Other medications may be used as adjuncts, such as diuretics, bronchodilators, and corticosteroids.^(40,41)^ The position of the patient with unilateral lung disease (shift to specific decubitus) can be an important non-pharmacological treatment.^(39)^

Oxygen is considered for patients with hypoxemia^(42)^ but some studies did not identify the benefit of oxygen compared to room air in non-hypoxemic patients.^(43)^

## ARTIFICIAL NUTRITION AND HYDRATION

Artificial nutrition and hydration (ANH) does not improve the outcomes for terminally ill patients and may, at times, increase patient distress. At that point, the ANH can cause nausea and increase the risk of aspiration.^(44)^

Artificial nutrition and hydration are medical interventions that can be withheld or withdrawn at any time during treatment. These decisions should be based on evidence, best practices, clinical experience, and judgment. Be sure to have an effective line of communication with the patient, family, and/or authorized surrogate decision maker, and respect for patient's autonomy and dignity.^(45-48)^

## DIALYSIS

In 2013, the total number of patients in Brazil with chronic renal disease under dialysis was 100,397.^(49)^ Many of these patients were treated in ICUs.

Nephrologists have a professional responsibility to understand the ethics behind medical decision-making and to intimately understand the well-defined recommendations for initiating and discontinuing dialysis. When nephrologists voice an alternative solution, everyone suffers less: patients, families, and even medical caregivers, including nephrologists.^(50)^ The Renal Physicians Association and the American Society of Nephrology recommend that clinicians should initiate timely and continuous discussion with dialysis patients and families to assist them in expressing their wishes about options in illness management at the end-of-life.^(51)^ Aiming to help with this difficult communication, Schell et al. described a communication skills workshop for nephrology fellows called NephroTalk.^(52)^

Once dialysis is initiated, nephrologists should evaluate the ongoing utility of it. The withdrawal of dialysis, when the globally established treatment is no longer beneficial for the patient, is currently a practice that is widely accepted in several countries.^(53)^

According to the second edition of the Renal Physician Association in regard to the decision of not initiating or discontinuing dialysis, the recommendations are the following: if appropriate, forgo (withhold initiating or withdraw ongoing) dialysis for patients with acute kidney disease (AKI), chronic kidney disease (CKD), or end-stage renal disease (ESRD) in certain, well-defined situations.

Withholding or withdrawing dialysis is appropriate in specific cases, including the following:

- Patients with decision-making capacity who, being fully informed and making voluntary choices, refuse dialysis or request dialysis to be discontinued.
- Patients who no longer possess decision-making capacity and have previously indicated refusal of dialysis in an oral or written advance directive.
- Patients who no longer possess decision-making capacity and whose properly appointed legal agents/surrogates refuse dialysis or request that it is discontinued.
- Patients with irreversible, profound, neurological impairment.

Medical management incorporating palliative care with attention to patient comfort and quality of life while dying should be addressed directly or managed by palliative care consultation and referral to a hospice program.

It is also recommended to consider forgoing dialysis for AKI, CKD, or ESRD patients who have a very poor prognosis or for whom dialysis cannot be provided safely. Included in these categories of patients are the following: those whose medical condition precludes the technical process of dialysis because the patient is unable to cooperate (e.g., an advanced dementia patient who pulls out dialysis needles) or because the patient's condition is too unstable (e.g., profound hypotension) or those who have a terminal illness from non-renal causes (acknowledging that some in this condition may perceive a benefit from and choose to undergo dialysis).

Forgoing dialysis should also be considered for those patients with stage 5 CKD who are older than 75 years of age and meet two or more of the following statistically significant very poor prognosis criteria:

1. Clinician's response of "No, I would not be surprised" to the "surprise" question, which means, in a multivariate analysis, the likelihood of death in 6 months was significantly greater when nephrologists answered no to the question, "Would I be surprised if this patient died within 6 months?"
2. High comorbidity score (e.g., modified Charlson Comorbidity index score of 8 or greater).
3. Significantly impaired functional status (e.g., Karnofsky Performance Status score less than 40).
4. Severe chronic malnutrition (i.e., serum albumin less than 2.5g/dL)

The ICU team must discuss these considerations with the nephrologists and try to reach a consensus. All providers are encouraged to participate in the decision-making process when dialysis is in question. A time-limited trial might be considered and then discussed with the family when a patient's prognosis is uncertain or if a consensus is not reached among the professionals.^(54-57)^

It has been suggested that when the decision to withdraw dialysis is made, both the patient and family should receive hospice services to provide emotional and spiritual support and to help manage physical symptoms. The most common symptoms in these patients are pain, uremic pruritus, sleep disorders, nausea, vomit, and constipation. Usually, these symptoms can be managed by a hospice service.^(55-59)^

## ADULT PALLIATIVE EXTUBATION

Another difficult decision to be made is about the withdrawal of mechanical ventilation. Intensivists could face a variety of situations in which patients should not be placed on artificial support due to a previous lack of communication on the goals of care, such as cases in which patients were intubated due to acute respiratory collapse and then transferred to the ICU. Most commonly, however, these situations arise due to the poor evolution of treatments associated with previous co-morbidities. Finally, those patients with a risk of developing a severe cognitive impairment should be considered.

In the last few years, there has been a growing acceptance that the withdrawal of mechanical ventilation can be part of palliative actions in the ICUs.^(60)^ In a study including Argentina, Brazil, and Uruguay, there were varying results about the suspension of mechanical ventilation. The authors identified these results as almost always held by 48.2% of Argentinian professionals, 25.8% of Uruguayan professionals, and 18.9% of Brazilian professionals.^(61)^ The bias of interviewing only participants in an event or active members of Intensive Care Societies might have influenced these results.

The following describes some important points for performing palliative extubation.

Practice the procedures to assure a patient's dignity:

1. Health team preparation:
  - Review the procedures to be performed in detail with the team standing by the patient.
  - All actions benefit the patient's dignity.
2. Prepare the patient and family:
  - Improve the time flexibility for the patient's visitors.
  - Discontinue unnecessary monitoring (such as cardiac monitor and pulse oximetry), treatments, and medications.
  - Ensure that the patient is calm and pain-free.

Practical procedures for palliative extubation:^(62)^

1. Withdraw enteral feeding 12 hours before extubation.
2. Withdraw neuromuscular blockers for at least two hours (note: in multiorgan failure, neuromuscular blockers act for up to 18 hours). Do not use neuromuscular blockers.
3. All staff who participate in the procedure must be close to the patient.
4. Be ensured that intravenous medications are being used to control the symptoms before and during extubation. The goal should be to relieve the symptoms, such as difficulty breathing and agitation.
5. Maintain venous access for administering medications to comfort the patient.
6. Keep a suction device for any oral secretion after extubation.
7. Raise bed headboard to 30 - 45 degrees.
8. Reduce FiO_2_ to room air and reduce the parameters by 50%. If the patient remains comfortable, reduce the pressure support and PEEP to assess the ventilation without discomfort. If the patient remains comfortable, perform extubation.
9. Use O_2_ mask for humidification after extubation.
10. Observe the symptoms of anxiety, dyspnea, and agitation, and treat them, if necessary.*

* Options medications to control symptoms.

Administer an IV bolus dose of an opioid and a benzodiazepine if anxiety is anticipated. Consider an IV continuous infusion of a sedating medication (see below). Do not rely on subcutaneous or enteral drug administration, as these take longer to work.

The most common symptoms related to the withdrawal of mechanical ventilation and the return to natural ventilation are agitation, breathlessness, and anxiety. Benzodiazepines and opioids are the medications used at this point.

Two drug options to control the symptoms in the adult palliative extubation are described below:

1. Morphine and Midazolam:^(62)^ good for comatose patients with decreased consciousness and/or patients who have received some of these medications previously.
  Bolus: Morphine 2 - 10mg; Midazolam 1 - 2mg
  Infusion: Morphine 50% of the of bolus in mg/h; Midazolam 1 mg/h
2. Propofol:^(62,63)^ appropriate for awake patients who may experience more pronounced respiratory distress after the discontinuation of mechanical ventilation.
  Bolus: 20 - 50mg
  Infusion: 10 - 100mg/h

The general process takes approximately 20 to 60 minutes. The presence of a multidisciplinary team is highly recommended to support the family with spiritual assistance, including a psychologist or a social worker.

The family should be aware of the possibility that, after the palliative extubation, the patient may remain in natural breathing for hours or days. In a recent study, half of the patients died after an hour of palliative extubation, most of them taking up to 10 hours. The dependence of Fi0_2_ greater than 70% and the use of vasoactive drugs were associated with a shorter time to the event of death.^(64)^

Note: If the advance directives of the patient's will are unknown and there is no designated representative or available family or there is a lack of consensus among them, the physicians must follow the Bioethics Committee of the Institution. Otherwise, the Regional Council of Medicine should be consulted for the advisement of proper procedures. This protocol is merely a suggestion. Each hospital should have its own protocol. The health team should use their clinical judgment and should not attempt the procedure if they are not prepared. Dosage ranges should be adjusted considering the patient's weight, hydration, and renal and hepatic function. Care should be taken if the patient is taking any concurrent medications. All variables should be considered.

## PALLIATIVE CARE AND HOSPICE

### Definition

Palliative care is for anyone in any stage with a serious illness and can occur along with curative treatment. It is not dependent on the prognosis and includes hospice care services. Therefore, in most clinical settings, it is delivered by the same group of healthcare professionals.

Hospice is an important Medicare benefit in the USA that provides palliative care for the terminally ill. People who receive hospice are also no longer receiving curative treatment for their underlying disease.

Hospice is considered the model for quality, compassionate care for people facing a life-limiting illness. In the USA, to qualify for hospice coverage under Medicare, a physician must confirm that the patient is expected to die within six months, should the patient's illness run the normal course. Hospice should provide expert medical care, pain management, and emotional and spiritual support that are expressly tailored to the patient's needs and wishes. Support is particularly provided to the patient's loved ones as well.

Hospice focuses on caring, not curing. In the USA, 80% of hospice care is provided in the patient's home. However, it may also be provided in hospitals or other facilities that specialize in taking care of patients in the last weeks or days of life. Usually, symptoms are controlled by a subcutaneous route. Hospice services are available for patients with any terminal illness, regardless of age, religion, or race.

Hospice care in the United States was established in the 1970s when cancer patients made up the largest percentage of hospice admissions. Even in the present day, cancer is still responsible for supplying the largest number of patients. The number of hospice programs nationwide continues to increase, with 6,100 programs today. In 2014, an estimated 1.6 to 1.7 million patients received services from hospice. The top four non-cancer primary diagnoses for patients admitted to hospice in 2014 were dementia (14.8%), heart disease (14.7%), lung disease (9.3%), and stroke or coma (6.4%).^(65)^

When the patient is transitioned to "Comfort Care," the ICU has the support of the palliative care team, which can include them in the care plan and also will follow the patient when they are discharged from the ICU.

## CONCLUSION

Mortality in intensive care units remains high, and the health team in intensive care units is constantly faced with complex situations where advanced treatment and advanced life support will not reach the goal of avoiding death, nor respect the patient and family's wishes. Discussion with the multidisciplinary team, as well as with the specialties involved in patient care, is critical. We must be prepared to discuss the limitations of technology to cure and provide comfort care with the patients and families. Many cases will require palliative care from a support team and advice from the hospital ethics committee. Hospitals should develop protocols for situations of conflict involving the specialties.
